# Enhanced Immobilization of Enzymes on Plasma Micro-Nanotextured Surfaces and Microfluidics: Application to HRP

**DOI:** 10.3390/molecules29194736

**Published:** 2024-10-07

**Authors:** Stefania Vorvi, Katerina Tsougeni, Angeliki Tserepi, Sotirios Kakabakos, Panagiota Petrou, Evangelos Gogolides

**Affiliations:** 1 Institute of Nanoscience & Nanotechnology, NCSR “Demokritos”, 15341 Aghia Paraskevi, Greece; svorvi@gmail.com (S.V.); k.tsougeni@gmail.com (K.T.); a.tserepi@inn.demokritos.gr (A.T.); 2 Immunoassays/Immunosensors Lab, Institute of Nuclear & Radiological Sciences & Technology, Energy & Safety, NCSR “Demokritos”, 15341 Aghia Paraskevi, Greece; skakab@rrp.demokritos.gr

**Keywords:** plasma nanotexturing, enzyme immobilization, enzymatic microreactor, horseradish peroxidase, 4-phenyl-azo-phenol oxidation

## Abstract

The enhanced and direct immobilization of the enzyme horseradish peroxidase on poly(methyl methacrylate) (PMMA) microchannel surfaces to create a miniaturized enzymatic reactor for the biocatalytic oxidation of phenols is demonstrated. Enzyme immobilization occurs by physical adsorption after oxygen plasma treatment, which micro-nanotextures the PMMA surfaces. A five-fold enhancement in immobilized enzyme activity was observed, attributed to the increased surface area and, therefore, to a higher quantity of immobilized enzymes compared to an untreated PMMA surface. The enzymatic reaction yield reached 75% using a flow rate of 2.0 μL/min for the reaction mixture. Additionally, the developed microreactor was reused more than 16 times without affecting the enzymatic conversion yield. These results demonstrate the potential of microchannels with plasma micro/nanotextured surfaces for the rapid and facile fabrication of microfluidic enzymatic microreactors with enhanced catalytic activity and stability.

## 1. Introduction

The term “immobilization of enzymes” was adopted in 1971 during the first Enzyme Engineering Conference and refers to the restriction of an enzyme to a phase (matrix/support) different from its substrate and the enzymatic reaction products [1]. The stability of immobilized enzymes and their ability to be reused were first explored by Grubhofer and Schelth, who studied the covalent binding of various enzymes such as carboxypeptidase, diastase, pepsin, and ribonuclease onto diazotized polyaminopolystyrene resin [2]. Since then, immobilized enzymes have had several applications, offering an enhanced level of control over the catalyzed reaction, reduced product contamination by the enzyme, and the suppression of undesirable side reactions [3,4]. However, the most significant advantage of immobilized enzymes is their ability to be reused without the need for complicated procedures, such as isolation from post-reaction mixtures. Additionally, with the careful selection of supporting materials and immobilization procedures, immobilized enzymes can be more stable and resistant to denaturation than enzymes in the liquid phase [5].

Although immobilized enzymes have been widely exploited in large-volume bioreactors for industrial applications, their incorporation into microfluidic devices has led to microfluidic immobilized enzyme reactors (μIMERs), which have been applied in numerous lab-scale processes ranging from proteomic studies to the development of biosensors [6,7,8,9,10,11,12,13,14,15,16,17]. The internal structure of these μIMERs typically falls into three categories: wall-coated, monolithic, and packed-bed types [10]. Wall-coated μIMERs offer the advantage of a low-pressure drop across the liquid flow and greater mechanical stability compared to the other two types. However, they are characterized by a lower surface-to-volume ratio, which negatively affects the yield of the enzymatic conversion.

An approach to increase the surface-to-volume ratio in wall-coated μIMERs is to structure the microreactor walls. Several methods can achieve this, including modifying the inner wall with nanostructured materials, such as dopamine, gold nanoparticles, graphene, graphene oxide, nanosprings, etc. [10,18]. Alternatively, increasing the enzyme loading onto the microreactor walls can be achieved by creating multiple immobilization layers through a process referred to as layer-by-layer assembly [19,20].

Another critical parameter that influences the performance of wall-coated μIMERs is the method of enzyme immobilization. Several techniques have been developed, including physical adsorption, covalent bonding, bio-affinity interaction, entrapment/encapsulation, and enzyme cross-linking [21,22,23]. The method of choice largely depends on the materials used to fabricate the μIMERs. Today, the most commonly used materials for enzyme microreactors are polymers such as polystyrene (PS) [24], poly(methyl methacrylate) (PMMA) [25], cyclo olefin copolymers [26], or silicon-containing polymers such as polydimethylsiloxane (PDMS) [22,27]. Polymeric surfaces are generally chemically inert and require chemical modification to permit enzyme immobilization. Common polymer treatment techniques employed for chemical activation include both wet and dry chemistry methods, such as UV irradiation, ozone, corona discharge, and plasma treatments [28,29].

Plasma processing is widely used to create functional groups such as NH_x_, C=O, COOH, and OH on polymeric surfaces by employing appropriate gas mixtures, which etch or deposit material on the surface [30]. Our group has pioneered plasma treatment procedures that achieve the chemical activation and micro-nanotexturing of polymeric surfaces [31,32,33,34]. These surfaces offer a higher biomolecule binding efficiency compared to planar surfaces due to an increased surface area [35], while also enabling covalent binding through appropriate surface chemistry [34]. Although these surfaces have been used as solid supports for protein microarrays [34], differential cell adhesion [36,37], and bacteria isolation [38], they have not yet been investigated as solid supports for microfluidic immobilized enzyme reactors.

**Figure 1:**
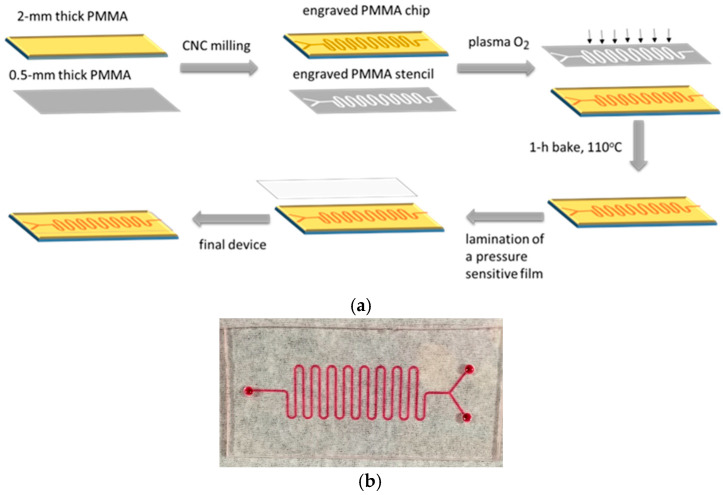
(**a**) Schematic presentation of microreactor fabrication procedure. (**b**) Image of the microreactor created on a 2 mm thick PMMA substrate prior to cover lamination (left) and of the stencil mask engraved on a 0.5 mm thick PMMA substrate (right). The stencil is placed on top of the microreactor during the plasma micro-nanotexturing step, as shown in (**a**).

In this work, a microfluidic reactor was fabricated on poly(methyl methacrylate) surfaces through micro-milling followed by treatment with oxygen plasma to introduce micro-nanotexturing, thereby increasing the surface area for enzyme immobilization (Figure 1). Horseradish peroxidase (HRP) was employed as a model enzyme, and different immobilization approaches were investigated to determine which provided the highest values in terms of immobilized enzyme quantity and activity. The latter was determined using an HRP precipitating substrate and quantifying the color developed through reflectance/absorbance measurements performed using an instrument equipped with an integrating sphere coupled to a spectrometer. Once the optimum enzyme immobilization conditions were established, HRP was immobilized onto the micro-nanotextured microreactor walls, and the catalytic oxidation of 4-phenylazophenol was monitored. This particular reaction was selected due to the increased interest in developing microreactors for the oxidation of phenolic compounds to less harmful substances [39,40,41]. To evaluate the enzymatic reaction yield, the microreactor outlet was fed into a flow cell connected to a light source and a spectrometer via optical fibers, and the light intensity at 347 nm, corresponding to the absorbance peak of 4-phenylazophenol, was monitored. The effects of several parameters, including the volume of the 4-phenylazophenol solution and its flow rate through the microreactor, on the enzymatic reaction yield were investigated, and the possibility of reusing the microreactor was also evaluated.

## 2. Results

### 2.1. Plasma Micro-Nanotexturing and Enzyme Immobilization on PMMA Open Surfaces

**Figure 2:**
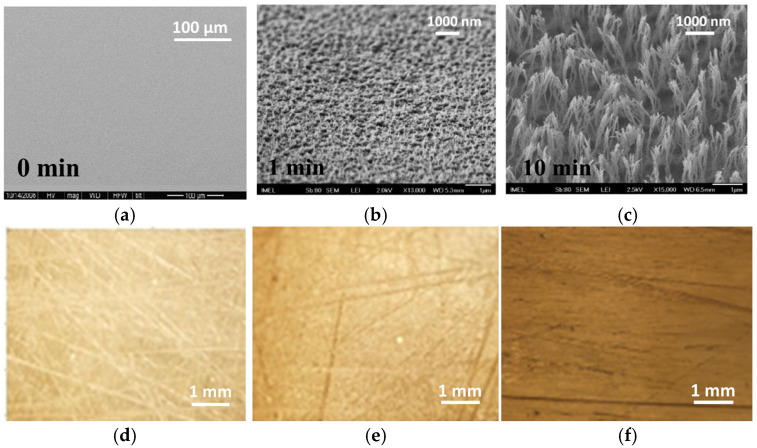
(**a**–**c**) SEM images from untreated (**a**), 1 min (**b**), and 10 min (**c**) oxygen-plasma-treated and annealed PMMA surfaces. Scale bars have been included to indicate the relative size of the micro/nanostructures created by the plasma treatment. (**d**–**f**) Optical microscope images from untreated (**d**), 1 min (**e**), and 10 min (**f**) oxygen-plasma-treated and annealed PMMA surfaces to which HRP has been immobilized after incubation with precipitating metal-enhanced DAB HRP substrate. Images were obtained using an optical microscope (AxioImager.A1m; Carl Zeiss, Hamburg, Germany) equipped with a 5× lens and a Sony Cybershot digital camera (Sony Group Corporation; Minato City, Tokyo, Japan).

Oxygen plasma etching in an RIE reactor was employed to micro-nanotexture PMMA surfaces, creating solid supports with an increased surface area for enzyme immobilization. The formation of this micro-nanotexture results from the deposition of silicon oxide particles on the surface, which act as local etching inhibitors. This leads to local variability in the etching rate and, consequently, the creation of surface roughness. The presence of silicon oxides on oxygen-plasma-treated polymeric materials is supported by XPS analysis results presented in previous publications [31,32,33,34]. SEM images of an untreated PMMA surface and PMMA surfaces treated with oxygen plasma for 1 and 10 min are shown in Figure 2. These SEM images demonstrate the progressive creation of micro-nanotexture on the initially flat PMMA surfaces (Figure 2a), resulting in micro-nano structures with heights of 0.3 μm (Figure 2b) and 1.3 μm (Figure 2c), respectively, as the duration of the oxygen plasma treatment increases from 1 to 10 min. The images of the untreated surfaces were obtained at a low magnification to demonstrate the smoothness of the surface, while the plasma-treated surfaces were imaged at a higher magnification to show the relative size and density of structures created. The immobilized enzyme activity was evaluated using a precipitating HRP substrate, the metal-enhanced DAB substrate. This HRP substrate contains cobalt chloride and nickel chloride in a special formulation that results in the formation of a dark brown/black precipitating product in the presence of HRP. First the incubation duration with the DAB HRP substate was optimized using 10 min oxygen-plasma-treated PMMA surfaces without HRP to determine the blank signal and with HRP to determine the specific signal. From the results presented in Figure S1, it can be concluded that the net signal (i.e., the signal from the HRP-coated surfaces minus that from the non-coated surfaces) increases as the incubation time increases from 5 to 15 min. When the incubation duration was extended to 30 min, the net signal remained the same as that for a 15 min incubation due to a considerable increase in the non-specific signal. Therefore, a 15 min incubation the DAB HRP substrate was selected for further experimentation.

Images of the colored precipitating product formed after HRP immobilization through adsorption onto untreated and oxygen-plasma-treated PMMA surfaces for 1 and 10 min are also provided in Figure 2. The images were acquired with an optical microscope at five times magnification to demonstrate the increase in color intensity, indicating a corresponding increase in the quantity of enzymes immobilized on the micro/nanotextured PMMA surfaces compared to the untreated ones. The optical microscope images also reveal the relatively homogeneous coverage of the surface by the precipitate from the enzymatic reaction, further indicating a uniform distribution of the enzyme across the surface.

**Figure 3:**
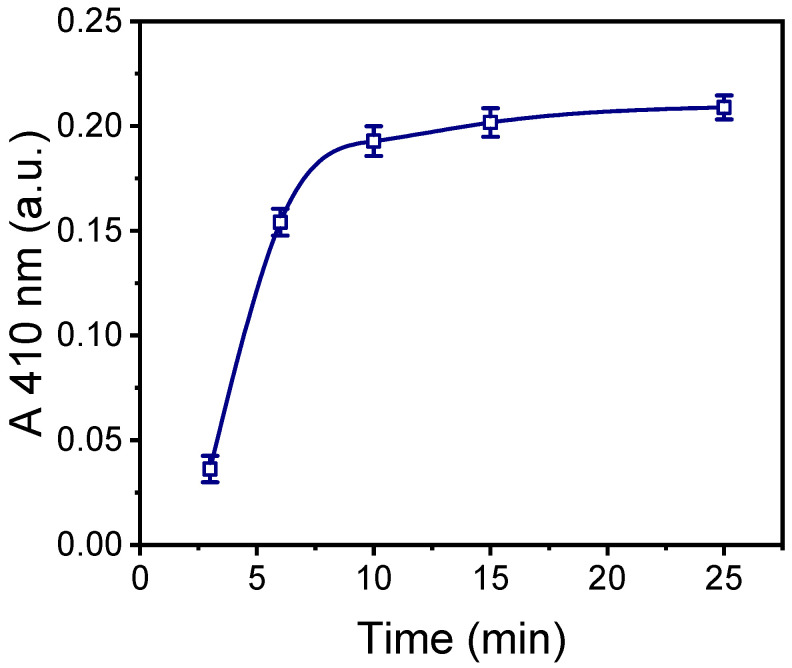
Absorbance values at 410 nm versus the duration of the PMMA surfaces’ exposure to oxygen plasma after incubation with a 200 μg/mL HRP solution at RT for 2 h. Immobilized HRP was detected through reaction with DAB precipitating HRP substrate for 15 min. Each point corresponds to mean value of triplicate measurements ± standard deviation.

The plasma processing duration was optimized based on the intensity of the colored layer formed by the precipitate deposited onto the surfaces as a result of the reaction between the immobilized enzyme and the DAB substrate. This intensity was measured using an integrating sphere and will be referred to as absorbance at 410 nm (A 410 nm). Figure 3 presents the absorbance at 410 nm as a function of the oxygen plasma treatment duration. As shown, maximum absorbance values were obtained from the surfaces treated with oxygen plasma for at least 10 min. Extending the plasma treatment beyond 10 min resulted in only a marginal increase in the precipitate intensity. Therefore, 10 min oxygen-plasma-treated surfaces were selected.

### 2.2. Selection of Method for HRP Immobilization to Oxygen-Plasma-Treated PMMA Surfaces

After selecting the oxygen-plasma-treated PMMA surface, the optimal HRP immobilization method was explored. In addition to physical adsorption, covalent bonding and affinity immobilization were tested. The covalent bonding method relied on the presence of carboxyl groups on the surface of the oxygen-plasma-treated PMMA, which were activated with a mixture of EDC/NHS to form active esters that could readily react with the amine groups on the HRP molecule. For the affinity-based immobilization approach, HRP was biotinylated to enable binding to PMMA surfaces modified with streptavidin.

**Figure 4:**
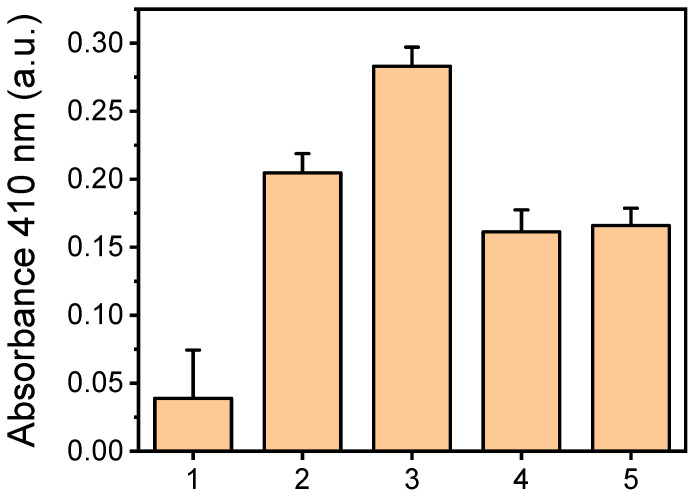
Absorbance values at 410 nm obtained from surfaces on which HRP has been immobilized through adsorption on untreated PMMA surfaces (column 1), adsorption on PMMA surfaces treated with oxygen plasma for 10 min (column 2), covalent binding on EDC/NHS-activated PMMA surfaces treated with oxygen plasma for 10 min (column 3), and affinity binding of biotinylated HRP on PMMA surfaces treated with oxygen plasma for 10 min and modified with 0.5 mg/mL (column 4) or 1.0 mg/mL streptavidin solution (column 5). All surfaces have been incubated for 15 min with the precipitating DAB HRP solution. Each point corresponds to mean value of triplicate measurements ± SD.

The absorbance values obtained from the PMMA surfaces treated for 10 min with oxygen plasma, with HRP immobilized through the three methods, are presented in Figure 4. For the affinity binding method, two concentrations of streptavidin were tested to ensure plateau values were reached. HRP was also immobilized by physical adsorption on untreated PMMA for comparison. As shown in Figure 4, the highest absorbance value was achieved with the oxygen-plasma-treated PMMA surfaces for which HRP was immobilized via covalent bonding. These values were four times higher than those for the untreated surfaces and 25% higher than those obtained from the oxygen-plasma-treated PMMA surfaces with HRP immobilized by physical adsorption. Compared to the untreated surfaces, the oxygen-plasma-treated ones provided a five-fold increase in signal for HRP immobilization through physical adsorption.

Affinity immobilization on the oxygen-plasma-treated surfaces yielded the lowest values compared to the other two methods. This result was somewhat unexpected, as affinity immobilization typically preserves enzyme activity better than physical adsorption or covalent bonding since the enzyme does not directly interact with the surface. However, it appears that lower amounts of enzyme were attached to the surface, likely due to steric hindrance that limited the access of biotinylated HRP to streptavidin binding sites. This was supported by the observation that the maximum plateau values were achieved for HRP concentrations of 100 μg/mL or higher with affinity immobilization, whereas physical adsorption and covalent bonding required HRP concentrations of 200 μg/mL or higher (Figure S2).

Another contributing factor may be the negative impact of biotinylation on enzyme activity, particularly if biotin moieties are located near the enzyme’s active site. Indeed, tests conducted in the liquid phase using a soluble HRP substrate (ABTS) confirmed that the activity of biotinylated HRP was 20% lower than that of non-biotinylated HRP. Although covalent bonding provided the highest signals, physical adsorption was selected for enzyme immobilization within the microreactor channel as the simplest and most cost-effective method.

### 2.3. Optimization of HRP Immobilization by Physical Adsorption

For the selected micro/nanotextured PMMA surfaces and HRP immobilization method, the duration of incubation with the HRP solution was determined. A 200 μg/mL HRP solution, which, according to the results presented in Figure S2, provided nearly the maximum plateau signal values, was used, and incubation times ranging from 1 to 18 h were tested. As shown in Figure S3, a 2 h incubation period with the HRP solution at room temperature (RT) was sufficient to achieve the maximum plateau signal values and was therefore chosen for enzyme immobilization through physical adsorption.

### 2.4. Comparison with the Untreated PMMA Surfaces

**Figure 5:**
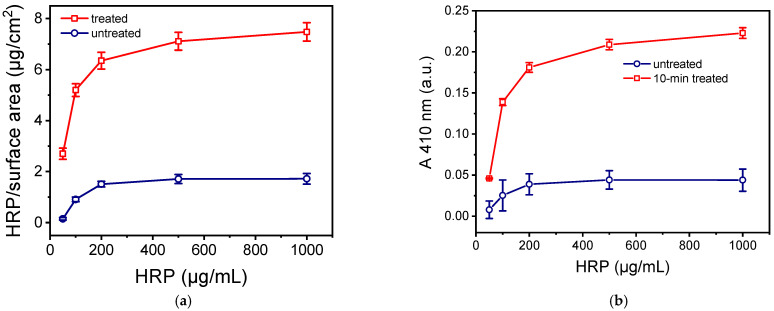
(**a**) HRP quantity per unit of surface area versus the HRP concentration in the coating solution determined for untreated (red circles) and PMMA surfaces treated with oxygen plasma for 10 min (black squares). The surfaces were incubated for 2 h with the HRP solutions and then for 15 min with the precipitating DAB HRP solution. Each point corresponds to mean value of triplicate measurements ± standard deviation. (**b**) Absorbance values at 410 nm obtained from untreated (blue circles) or PMMA surfaces treated with oxygen plasma for 10 min (red squares) versus the HRP concentration. The duration of incubation of the surfaces with the HRP solution was 2 h. Immobilized HRP was detected in all cases through reaction with DAB precipitating HRP substrate for 15 min. Each point corresponds to mean value of triplicate measurements ± standard deviation.

The selected micro/nanotextured PMMA surfaces were compared to the untreated ones in terms of their capacity for HRP immobilization by physical adsorption and the enzymatic activity of the immobilized HRP. Both types of surfaces were coated with HRP solutions at concentrations ranging from 50 to 1000 μg/mL for 2 h. The amount of immobilized enzyme was determined using the BCA method, while the enzymatic activity of the immobilized enzyme was assessed through a reaction with the DAB HRP substrate. As shown in Figure 5a, which presents the density of immobilized HRP per unit of surface area relative to the HRP concentration in the coating solution, the values obtained across the entire concentration range tested on the oxygen plasma micro/nanotextured surfaces were five times higher than those observed on the untreated surfaces. Moreover, while the untreated surfaces appeared to saturate at a 200 μg/mL HRP concentration, the oxygen-plasma-treated surfaces showed a slight increase in signal with higher HRP concentrations. A similar trend is observed when the enzymatic activity of the immobilized enzyme was measured (Figure 5b). Additionally, the five-fold difference in enzymatic activity between the oxygen-plasma-treated and untreated surfaces indicates that the plasma-treated surfaces immobilized five times more HRP than the untreated ones.

### 2.5. Evaluation of Plasma-Nanotextured Microreactor Performance

The evaluation of the HRP microreactor was conducted through the catalytic oxidation of 4-phenylazophenol. For this purpose, mixtures of 4-phenylazophenol and H_2_O_2_ were introduced into the microreactor at a constant flow rate, and the catalytic reaction efficiency was assessed by recording the absorbance peaks at 347 nm of the solution exiting the microreactor after reacting with the immobilized HRP. A total sample volume of 3 μL was introduced into the microreactor.

**Figure 6:**
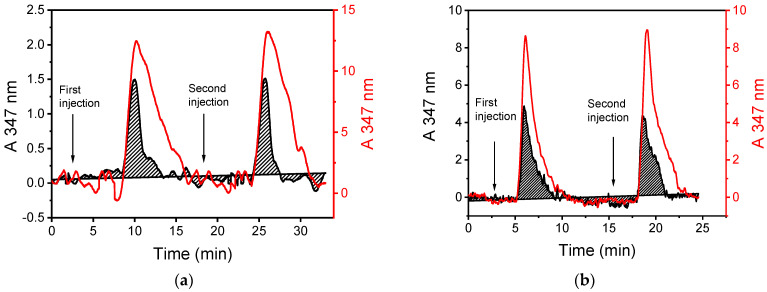
Absorbance profiles at 347 nm obtained upon running a 1:1.5 *v*/*v* 4-phenylazophenol/H_2_O_2_ mixture at flow rates of (**a**) 2.0 μL/min and (**b**) 7.5 μL/min through a chip microreactor treated with oxygen plasma for 10 min without (red line) and with immobilized HRP (black shaded graph). The time of each injection is indicated by arrows. Two injections with 3 μL of the 1:1.5 4-phenylazophenol/H_2_O_2_ mixture were performed at the indicated time points.

The most critical parameter for optimizing the microreactor’s catalytic activity was the flow rate of the 4-phenylazophenol/H_2_O_2_ mixture. Flow rates ranging from 2.0 to 30 μL/min were tested. Figure 6 shows the elution profiles of the mixture at flow rates of 2.0 μL/min (Figure 6a) and 7.5 μL/min (Figure 6b) of both the non-modified (red line) and HRP-modified microreactors (black line). In all cases, two sequential injections were used. The maximum absorbance peak was detected 6.0 and 2.5 min after each injection for flow rates of 2.0 μL/min and 7.5 μL/min, respectively. Additionally, Figure 6 clearly shows that the absorbance peak area decreased when the reaction mixture passed through the plasma micro/nanotextured microreactor modified with HRP compared to when it passed through a microchannel that was not modified with the enzyme.

The absorbance peak area value of the HRP-modified microreactor (*P_HRP_*) and the respective peak area value of the non-modified microreactor (*P_NOHRP_*) were used to calculate the conversion percentage of 4-phenylazophenol for each tested flow rate. The conversion percentage was determined using the following equation:

$$\%Conversion=1-\left[\frac{P_{HRP}}{P_{NOHRP}}\right]\times 100$$

**Figure 7:**
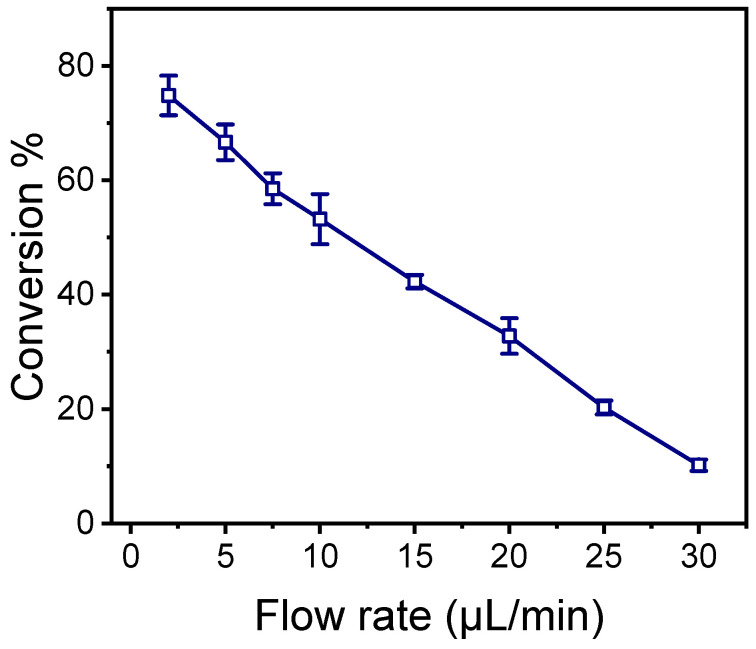
Conversion percentage of 4-phenylazophenol versus the flow rate of the reaction mixture through an oxygen-plasma-treated HRP-modified microreactor. The conversion is calculated using an oxygen-plasma-treated chip without HRP as reference. Each point is the mean value of triplicate measurements ± standard deviation.

The conversion percentage values as a function of the HRP substrate flow rate are presented in Figure 7. As expected, the conversion percentage was inversely proportional to the flow rate; the highest conversion percentage was achieved at the lowest flow rate, with 75% conversion at 2.0 μL/min. At the highest flow rate tested (30 μL/min), the conversion percentage was less than 10%.

Another parameter expected to affect the performance of the enzymatic microreactor is the sample volume. The sample volume cannot exceed the total volume of the microreactor channel, which is 16 μL. It was observed that increasing the sample volume from 3 μL to 6 μL and then to 10 μL resulted in a decrease in the conversion percentage, from 75% to 40% and then to 20%, respectively. Therefore, to achieve the highest conversion percentage, the sample volume should be kept as low as possible.

The possibility of reusing the HRP-modified microreactor was also assessed. Figure 6 shows the results of two injections of the 4-phenylazophenol/H_2_O_2_ mixture, indicating that there is no significant loss in the activity of the immobilized enzyme, allowing the microreactor to be used multiple times. Specifically, it was found that a single microreactor could be reused at least 16 times without a statistically significant effect on the conversion percentage of 4-phenylazophenol (Figure S4).

Additionally, the stability of the enzyme immobilized on the oxygen plasma micro/nanotextured surface was evaluated. For this purpose, microreactors modified with HRP were stored in a refrigerator at 4 °C under a controlled level of humidity for 15 days before testing their ability to convert 4-phenylazophenol. Compared to the catalytic activity of microreactor chips used immediately after HRP immobilization, the catalytic activity of the chips after 15 days of storage ranged from 84% to 89%.

## 3. Discussion

The performance of an enzyme microfluidic reactor is primarily defined by two parameters: its microfluidic design and the immobilization of the enzyme [10]. From the perspective of microfluidic design, the serpentine channel adopted in this work offers the advantage of increasing the microchannel surface area without enlarging the device’s overall footprint, as would be the case with a straight microchannel. To further enhance the performance of the designed enzyme microfluidic reactor, the bottom of the channel was micro/nanotextured via oxygen plasma treatment, a process that has been shown to create brush-like structures. These structures can reach lengths of several micrometers while having widths of only a few nanometers [31,32,33]. The width of the structures is determined by the size of silicon oxide particles sputtered onto the substrate during exposure to oxygen plasma, while the length increases with treatment time (Figure 2), leading to an increase in the active surface area. However, there is a point at which these structures begin to collapse and merge, resulting in the stabilization of or even the reduction in the available surface area [33,34]. For the substrate and biomolecule used in this study, this point is reached after 10 min of the oxygen plasma treatment. At this stage, compared to the untreated surfaces, the PMMA surfaces treated with oxygen plasma for 10 min exhibit an approximately five times greater active surface area [33,34]. This increase in the active-to-projected surface area ratio is reflected in both the amount of HRP immobilized and the activity of the immobilized enzyme.

Another critical parameter influencing the performance of enzymatic microfluidic reactors is the method of enzyme immobilization [10,21,22,23]. Among the various methods available, this study explores physical adsorption, covalent bonding, and affinity immobilization through the biotin–streptavidin system. Covalent bonding primarily depends on the carboxyl groups formed on the PMMA structures during the oxygen plasma treatment and requires the transformation of these groups into active esters to enable their reaction with the free amine groups on the enzyme molecules. Physical adsorption, on the other hand, is simpler since it does not require additional surface treatment. Furthermore, based on our previous findings, oxygen plasma treatment endows the PMMA surface with highly reactive carbonyl groups that may also contribute to enzyme immobilization. However, it is challenging to experimentally determine the extent to which covalent bonding contributes to enzyme attachment. This combination of physical adsorption, which is known to induce changes in protein conformation, and covalent bonding could explain why the enzymatic activity of the physically adsorbed enzyme was 25% lower than that of the covalently attached enzyme. An unexpected finding was that affinity binding through the biotin–streptavidin system resulted in significantly lower enzymatic activity (almost 50% less than that of covalent bonding), despite the expectation that this method would better preserve the enzyme’s native configuration and activity. This reduced activity, along with the observation that the amount of biotinylated enzyme required to reach the maximum plateaued signal was half that of the amounts required for physical adsorption and covalent bonding, suggests that a smaller quantity of a less active enzyme was immobilized using this approach. The loss of enzyme activity could be attributed to biotinylation near the enzyme’s active site. Despite the higher immobilized enzyme activity achieved by covalent bonding, physical adsorption was selected for the continuation of the study due to its simplicity.

The final evaluation of the HRP microreactor was performed by running a mixture of 4-phenylazophenol and H_2_O_2_ at a constant flow rate while monitoring the catalytic reaction by spectroscopically detecting the enzymatic reaction product at the microfluidic outlet. As expected, the yield of the enzymatic reaction was influenced by the flow rate of the reaction mixture. The highest enzymatic conversion rate (approximately 75%) was achieved at the lowest flow rate used (2.0 μL/min). This conversion rate is slightly lower than those reported for other enzymatic microreactors in the literature, according to which conversion rates of up to 90% have been noted [10]. It is important to mention that these microreactors use different enzymes and have very different configurations compared to the one proposed here. Lower flow rates could potentially increase the enzymatic conversion rate further, but with the experimental setup used, it was not possible to maintain a stable flow at lower rates. Moreover, at lower flow rates, the increased time required to run the sample through the microreactor would counterbalance the increase in enzymatic conversion rate. From a performance perspective, the possibility of reusing the proposed enzymatic microreactor without a decline in the conversion rate is also of great importance. Although the microreactor was tested only 16 times, the fact that the enzyme activity did not deteriorate suggests that both the amount and activity of the immobilized enzyme are well preserved. In summary, the simple fabrication method, direct enzyme immobilization, stable enzymatic activity, and high conversion rate are the key advantages of the enzymatic microreactor presented in this work.

## 4. Material and Methods

### 4.1. Materials

Poly(methyl methacrylate) (PMMA) plates that were 2 mm thick were purchased from IRPEN (Alcalá de Henares, Spain). Horseradish peroxidase (HRP, VI-A), 2,2′-azino-bis(3-ethylbenzothiazoline-6-sulfonic acid) diammonium salt (ABTS), and 4-phenylazophenol were purchased from Sigma-Aldrich (Darmstadt, Germany). ImmunoPure^®^ metal enhanced DAB peroxidase substrate working solution, Pierce™ BCA Protein Assay Kit, 1-ethyl-3-[3-dimethylaminopropyl]carbodiimide hydrochloride (EDC), N-hydroxysulfosuccinimide (sulfo-NHS), ImmunoPure^®^ streptavidin, and sulfosuccinimidyl-6-[biotin-amido]hexanoate (sulfo-NHS-LC-biotin) were purchased from Thermo Fisher Scientific Inc. (Waltham, MA, USA). All other reagents were of analytical grade and were purchased from Merck KGaA (Darmstadt, Germany).

### 4.2. Fabrication of Open PMMA Surfaces

The PMMA plates were cut in pieces with diameter of 4.5 × 5.5 cm^2^ and ultrasonically cleaned in isopropyl alcohol and deionized water. On each piece, six wells were fabricated by hot embossing in a hydraulic press (Carver model 3850 CE) using an aluminum stamp with six pillars with dimensions of 5 mm × 5 mm × 1.5 mm (LxWXH). The embossing was performed by applying pressure of 140 kp/cm^2^ at a temperature of 135 °C for 20 min, followed by slow cooling to room temperature (RT) prior to PMMA plates being separated from the aluminum stamp. The wells formed this way had capacity of 37.5 μL. A schematic of the open plasma nanotextured PMMA surfaces’ fabrication procedure is provided in Figure S4.

Oxygen plasma processing was performed in a Nextral reactive ion etching (RIE) reactor under anisotropic etching conditions (plasma power of 400 W at 13.56 MHz and pressure of 10 mTorr). After that, a thermal annealing step was performed at 110 °C for 1 h to stabilize the contact angle while preserving most of the carbonyl and carboxyl functional groups [34]. Water contact angles were measured in triplicate at ambient atmospheric conditions using 5 μL water drops in a GBX-DIGIDROP apparatus. An FEG-SEM (samples viewed at 80° tilt) was used for surface topographical characterization of the plasma-treated surfaces. The height of the structures formed was calculated by the instrument software from cross-view images obtained at the edge of the substrates.

### 4.3. Microreactor Fabrication Process

The microreactor consists of a channel with 17 meanders with depth of 158± 30 μm, width of 500 μm, total length of 8 cm, and volume capacity of 16 μL, fabricated on 2 mm thick PMMA sheets by micromilling. The PMMA sheet with the milled microreactor is subjected to a 10 min long oxygen plasma processing step through a stencil mask prepared on 0.5 μm thick PMMA sheets. Thus, upon exposure to oxygen plasma, the surface roughening and functionalization are restricted inside the microchannel, while the surface outside the microchannel remains smooth to facilitate sealing by lamination of a pressure-sensitive-adhesive film. After plasma exposure, an annealing step at 110 °C for 1 h is performed. The microreactor fabrication process is schematically depicted in Figure 1a, and an image of the actual microreactor and the PMMA stencil mask are provided in Figure 1b.

### 4.4. Immobilization of HRP on Open PMMA Surfaces

#### 4.4.1. Physical Adsorption

Immobilization of HRP on PMMA surfaces (untreated, oxygen plasma treated, and oxygen plasma treated and annealed) by physical adsorption was performed by manual deposition of 25 μL of a 200 μg/mL HRP solution in 100 mM phosphate buffer (pH 7.4) and incubation at RT in a humidity chamber. After that, the surfaces were washed three times with 100 mM phosphate buffer at a pH of 7.4 (washing buffer) to remove any unbound enzyme from the surface, prior to determination of immobilized enzyme activity.

#### 4.4.2. Covalent Bonding through EDC/NHS

For the covalent immobilization of HRP on the oxygen-plasma-treated PMMA surfaces, activation of the carboxyl groups was performed through incubation with a mixture containing 10 mM EDC and 5 mM sulfo-NHS in 0.1 M MES buffer at a pH of 5.0 for 1 h at RT. After washing them to remove excess reagents, the surfaces were incubated for 2 h in a humidity chamber with a 200 μg/mL HRP solution in 100 mM phosphate buffer at a pH of 7.4. The surfaces were washed with washing buffer and distilled water and dried under a N_2_ stream prior to the determination of immobilized enzyme activity.

#### 4.4.3. Affinity Immobilization via Streptavidin

In order to immobilize HRP via streptavidin, the enzyme was first biotinylated as follows: a 100 μg/mL sulfo-NHS-LC-biotin solution was prepared in dimethyl-sulfoxide and added drop-wise to a 2 mg/mL HRP solution in 0.25 carbonate buffer at pH of 9.1 containing 9 g/L ΝaCl. The mixture was incubated for 2 h at RT and then extensively dialyzed against a 0.1 M NaHCO_3_ solution at a pH of 8.5 with 9 g/L NaCl to remove the excess biotin. The biotinylated HRP solution was then kept at 4 °C.

PMMA surfaces were incubated with a 500 μg/mL streptavidin solution in 100 mM phosphate buffer at pH of 7.4 for 2 h at RT, followed by immersion in a 10 g/L bovine serum albumin (BSA) solution in the same buffer (blocking solution) for 1 h at RT, in order to cover the remaining free binding sites of the surface. After that, the surfaces were washed and a 100 μg/mL biotinylated HRP solution, prepared in blocking solution, was added and incubated for 1 h at RT. The surfaces were washed and dried as described previously prior to determination of immobilized enzyme activity.

### 4.5. Evaluation of Immobilized Enzyme Activity of Open PMMA Surfaces

The immobilized HRP enzymatic activity was determined through reaction with the ImmunoPure^®^ metal-enhanced DAB HRP substrate working solution for 15 min at room temperature (RT). Subsequently, the surfaces were washed three times with washing buffer and distilled water and dried under a stream of N_2_. The reaction resulted in a dark brown/black precipitate, the intensity of which depends on the amount of enzyme immobilized on the surface (Figure S1). To quantify the intensity of the brown/black precipitate, reflectance/absorbance measurements were performed using an FR-Reflection instrument (ThetaMetrisis S.A.; Athens, Greece) equipped with an Ocean Optics ISP-50-GT Integrating Sphere coupled to an Ocean Optics QE65000-ABS UV-NIR spectrometer. For the measurements, the surface was placed at the sphere port, and the incident light flux (with the light excitation angled at 8°) was reflected by the sample. The sphere collected the total reflectance from the top rough micro-nanotextured surface, while the transmitted light passed through the sample and was reflected by the silicon wafer on which the surface was placed. The silicon wafer had the role of a reference mirror surface with R = 46.7% at 410 nm. The light reflected from the mirror entered through the micro-nanotextured top surface back into the integrating sphere. Thus, the sphere collected both the light reflected from the precipitate and the largest part of the transmitted light through the precipitate. Therefore, the signal recorded was related to the absorbance of the insoluble product from HRP reaction with the DAB substrate formed on each surface. An oxygen-plasma-treated sample without enzyme was used as reference surface. Using the method described, reflectance-enhanced absorbance (*A*) was calculated using the following equation:

$$A=log(\frac{Isample-Idark}{Iref.-Idark})$$

where *Iref.* is the reference light intensity (i.e., from the sample without enzyme), *Isample* is the light intensity reflected from the sample with enzyme, and *Idark* is the signal intensity recorded by the spectrometer with the light source switched off and without any samples (background signal). The integration time for each measurement was 4 min.

### 4.6. Evaluation of Immobilized Enzyme Quantity

The quantity of the enzyme immobilized on PMMA surfaces was performed using the BCA Protein Assay Kit following the manufacturer’s instructions. Briefly, a working solution was prepared by mixing 50 parts of BCA reagent A with 1 part of BCA reagent B. Then, 30 μL of this solution was applied to the wells formed by hot embossing of PMMA surfaces to which HRP was immobilized and incubated for 2 h at RT in a humidity chamber. From each well, 25 μL was collected and transferred to a microtiter plate for measurement of the optical density at 560 nm. The absorbance signal was correlated to the amount of immobilized enzyme through a calibration curve obtained with HPR solutions.

### 4.7. Enzyme Immobilization on Oxygen-Plasma-Treated Microchannels and HRP Activity Determination

Immediately after oxygen plasma treatment and annealing, the immobilization of HRP on the rough microchannel of the microreactor was performed by pipetting a 200 μg/mL HRP solution in 100 mM phosphate buffer at a pH of 7.4 and incubating it overnight at room temperature in a humidity chamber. Subsequently, the microchannel surfaces were extensively washed with washing buffer and distilled water and dried under a N_2_ stream. For comparison, untreated and plasma-treated PMMA chips without HRP were used as reference. After that, the microchannel was closed by lamination of a pressure-sensitive adhesive film and connected to Labsmith pumps and valves, as shown in detail in Figure S6, to determine the activity of HRP-modified microreactor regarding the catalytic oxidation of 4-phenylazophenol. For this purpose, a 1:1.5 molar ratio mixture of 4-phenylazophenol to 30% H_2_O_2_ solution was prepared. The experimental setup implemented two syringe pumps, one for continuous delivery of the mobile phase, i.e., the 100 mM phosphate buffer (pH 7.4) and the second for injection of the enzyme substrate. Three micro-valves, connected to each other and to the chip via PEEK capillary tubes, regulated the reagent sequence. The stream exiting the central microvalve was connected to the chip through a micromixer that split the flow of the reaction mixture into the two entrances of the chip. As shown, the outlet stream of the chip was introduced into a Z-type flow cell, connected to the light source and the spectrometer through optical fibers. As the incident light passes through the solution inside the flow cell, the spectrometer records the light intensity at 347 nm that corresponds to the absorbance peak of 4-phenylazophenol. The degree of phenol oxidation is calculated by the absorbance recorded prior to and after the enzymatic reaction.

## 5. Conclusions

Polymeric surfaces were micro-nanotextured using oxygen plasma to increase the surface area, creating surfaces suitable for an enzymatic microreactor through the immobilization of horseradish peroxidase (HRP). Open surfaces prepared using the same method were used to determine the optimal conditions for micro/nanotexturing and enzyme immobilization. Micro-nanotexturing resulted in a five-fold increase in HRP enzymatic capacity compared to that of the untreated surfaces, indicating a five-fold higher quantity of HRP enzymes immobilized on the treated surfaces. This result is in agreement with the five-fold surface area increase due to micro-nanotexturing created by the plasma treatment. The micro-nanotextured surfaces were incorporated into a microreactor and evaluated for their performance in the biocatalytic oxidation of phenols, specifically 4-phenylazophenol. The highest conversion ratio of 75% was achieved at the lowest flow rate of the reaction mixture, demonstrating the efficiency of the HRP-modified PMMA microreactor for phenol removal from aqueous solutions. Furthermore, the enzyme immobilized on the microreactor’s micro-nanotextured surface maintained its catalytic activity over multiple reaction cycles. These results highlight the potential of plasma micro/nanotextured surfaces for the rapid and facile fabrication of biocatalytic microfluidic devices.

## Data Availability

The data presented in this study are available upon request from the corresponding author. The data are not publicly available due to privacy issues.

## Supplementary Materials

The following supporting information can be downloaded at: https://www.mdpi.com/article/10.3390/molecules29194736/s1, Figure S1. Absorbance values at 410 nm obtained from PMMA surfaces treated with oxygen plasma for 10 min not coated with HRP (orange columns) or coated with a 100 μg/mL HRP solution as a function of incubation duration with the DAB HRP substrate. Each column corresponds to mean value of triplicate measurements ± SD; Figure S2. Absorbance values at 410 nm obtained from surfaces to which HRP has been immobilized on 10-min oxygen plasma-treated PMMA surfaces through: adsorption (column 1), covalent binding (column 2) or affinity binding of biotinylated HRP on surfaces modified with a 0.5 mg/mL streptavidin solution. Each point corresponds to mean value of triplicate measurements ± SD; Figure S3. Absorbance values at 410 nm obtained from 10-min oxygen plasma treated PMMA versus the duration of incubation with a 200 μg/mL HRP solution. All surfaces have been incubated for 15 min with the precipitating DAB HRP solution. Each point corresponds to mean value of triplicate measurements ± standard deviation; Figure S4. Values of %conversion of 4-phenylazophenol obtained from an HRP-modified oxygen-plasma micro/nanotextured PMMA microreactor upon 16 sequential injections of 4-phenylazophenol/H_2_O_2_ mixtures. The red line corresponds to the mean of the 16 values and the blue lines to mean ± 2 SD of the 16 values; Figure S5. Schematic of the procedure for fabrication of open PMMA oxygen plasma nanotextured surfaces and of the procedure for HRP immobilization and detection on them; Figure S6. (a) Schematic of pumps and valves connection to the chip placed on a dedicated holder. (b) Image of the chip placed on the dedicated holder and connected to the valves and pumps that regulate the flow of the running buffer and the sample.
