# Supplementary material for: Enhanced Immobilization of Enzymes on Plasma Micro-Nanotextured Surfaces and Microfluidics: Application to HRP
Source: Molecules. 2024 Oct 7;29(19):4736. doi: 10.3390/molecules29194736 (PMC11477641; doi:10.3390/molecules29194736)
Supplement: Supplementary file 1 [file molecules-29-04736-s001.zip › molecules-3184700-supplementary.pdf]

molecules

MDPI

Supplementary material

Enhanced Immobilization of Enzymes on Plasma Micro-Nano-textured Substrates and Microfluidics: Application to HRP

Stefania Vorvi[1], Katerina Tsougeni[1], Angeliki Tserepi[1], Sotirios Kakabakos[2], Panagiota Petrou[2] and Evangelos Gogolides[1]

[1] Institute of Nanoscience & Nanotechnology, NCSR "Demokritos", 15341 Aghia Paraskevi, Greece; svorvi@gmail.com (S.V.); k.tsougeni@gmail.com (K.T.); a.tserepi@inn.demokritos.gr (A.T.);

[2] Immunoassays/Immunosensors Lab, Institute of Nuclear & Radiological Sciences & Technology, Energy & Safety, NCSR "Demokritos", 15341 Aghia Paraskevi, Greece; skakab@rrp.demokritos.gr (S.K.)

* Correspondence: ypetrou@rrp.demokritos.gr (P.P), +30 210 6503819; e.gogolides@inn.demokritos.gr (E.G.), +30 210 650 3307

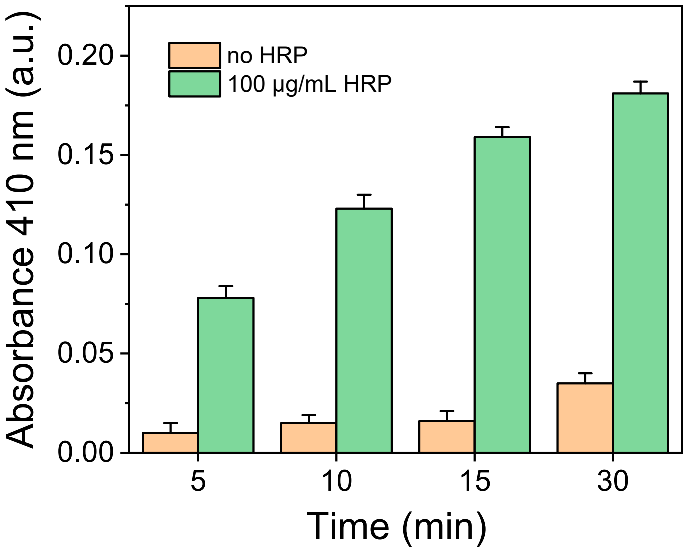

Figure S1. Absorbance values at 410 nm obtained from PMMA surfaces treated with oxygen plasma for 10 min not coated with HRP (orange columns) or coated with a 100 μg/mL HRP solution as a function of incubation duration with the DAB HRP substrate. Each column corresponds to mean value of triplicate measurements ± SD.

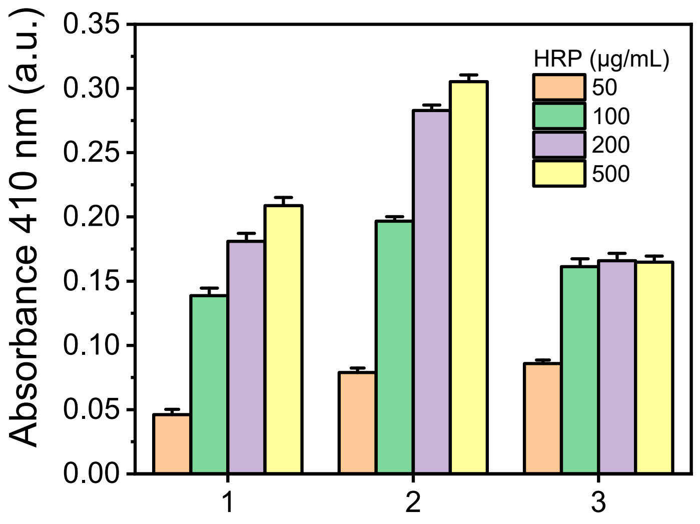

Figure S2. Absorbance values at 410 nm obtained from surfaces to which HRP has been immobilized on 10-min oxygen plasma-treated PMMA surfaces through adsorption (column 1), covalent binding (column 2) or affinity binding of biotinylated HRP on surfaces modified with a 0.5 mg/ml streptavidin solution. Each point corresponds to mean value of triplicate measurements ± SD.

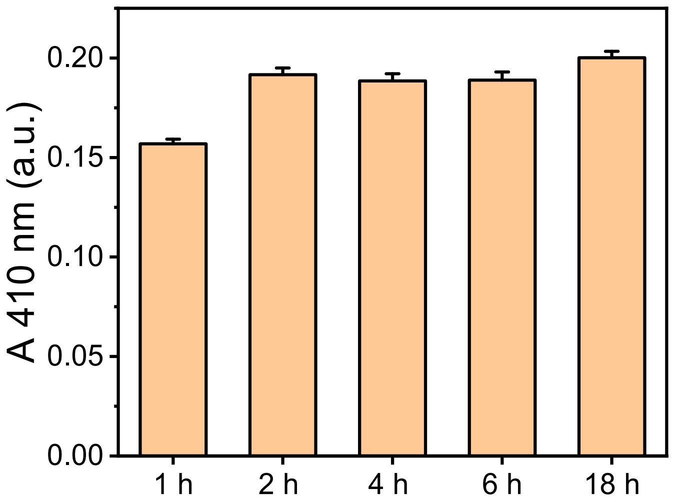

Figure S3. Absorbance values at 410 nm obtained from 10-min oxygen plasma treated PMMA versus the duration of incubation with a 200 μg/mL HRP solution. All surfaces have been incubated for 15 min with the precipitating DAB HRP solution. Each point corresponds to mean value of triplicate measurements ± standard deviation.

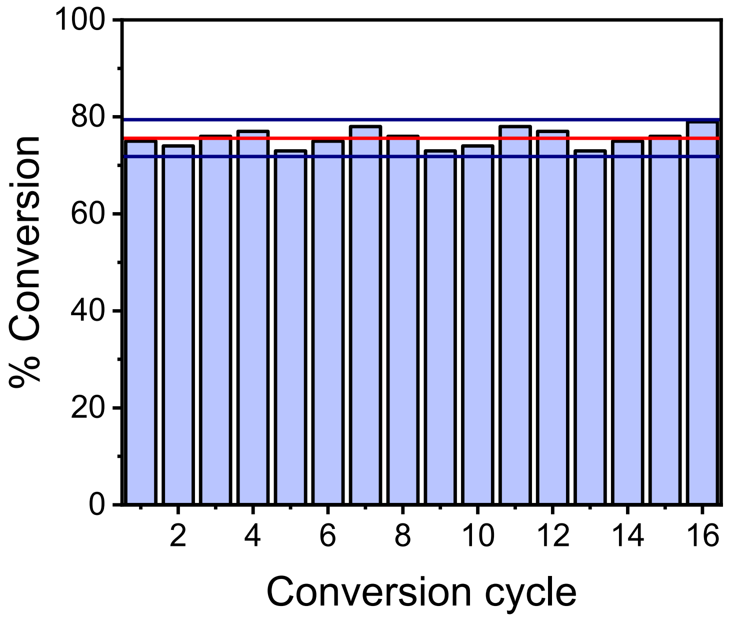

Figure S4. Values of %conversion of 4-phenylazophenol obtained from an HRP-modified oxygen-plasma micro/nanotextured PMMA microreactor upon 16 sequential injections of 4-phenylazophenol/H_2O_2 mixtures. The red line corresponds to the mean of the 16 values and the blue lines to mean±2SD of the 16 values.

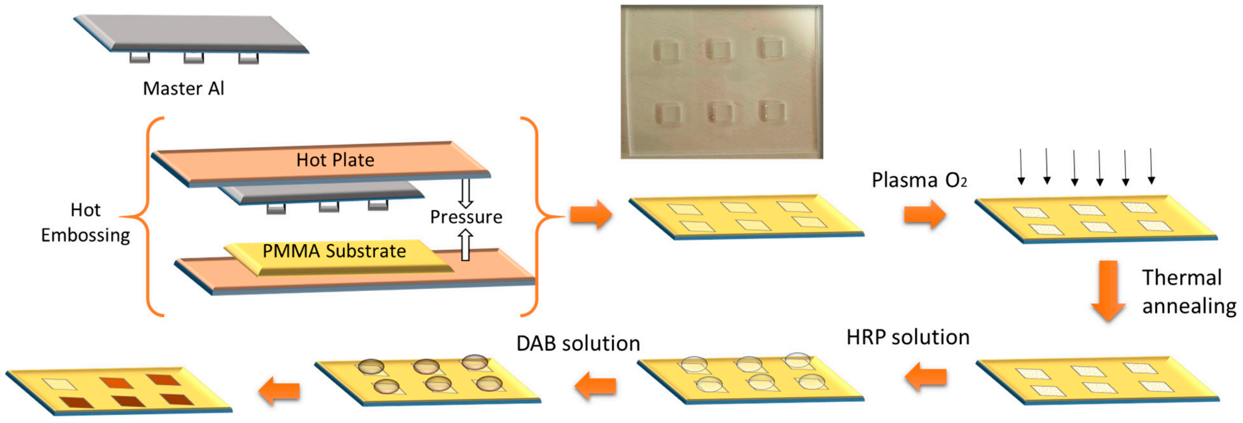

Figure S5. Schematic of the procedure for fabrication of open PMMA oxygen plasma nanotextured surfaces and of the procedure for HRP immobilization and detection on them.

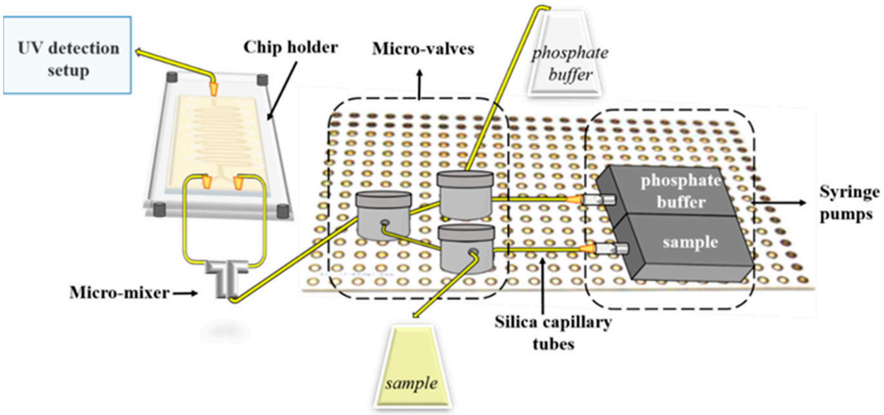

(a)

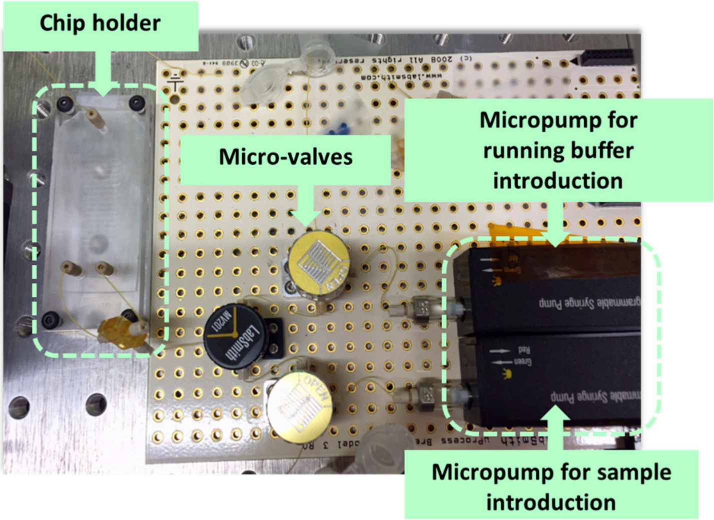

(b)

Figure S6. (a) Schematic of pumps' and valves' connection to the chip placed on a dedicated holder. (b) Image of the chip placed on the dedicated holder and connected to the valves and pumps that regulate the flow of the running buffer and the sample.
